# Public Health Challenges and Responses to the Growing Ageing Populations

**DOI:** 10.1002/puh2.213

**Published:** 2024-07-21

**Authors:** Hafiz T. A. Khan, Kwaku Mari Addo, Helen Findlay

**Affiliations:** ^1^ Public Health Group College of Nursing Midwifery and Healthcare University of West London London UK; ^2^ Oxford Institute of Population Ageing University of Oxford Oxford UK; ^3^ Ghana Ministry of Health Accra Ghana; ^4^ Independent Researcher

**Keywords:** ageing populations, challenges, global, public health, responses

## Abstract

**Background:**

Human populations are rapidly ageing worldwide due to declining birth rates and rising life expectancies. This profound demographic shift presents complex public health challenges. Synthesizing evidence on key public health issues impacting ageing populations and policy strategies is required to address these needs.

**Methods:**

The study employs narrative literature review based on the PubMed database. Data have been extracted on public health challenges to ageing populations and its recommended policy solutions.

**Results:**

The key public health challenges identified include rising chronic disease burden, risks for preventable multi‐morbidities and co‐morbidities, disability and dependencies, mental health issues, caregiving gaps, long‐term care system deficiencies, health inequities, healthcare access barriers, end‐of‐life care needs, financial instability, ageism/elder abuse, adverse built environments, climate/disaster threats, and social isolation. Evidence‐based policy responses span interventions in healthcare, social services, urban planning, emergency preparedness, economics, technology, anti‐ageism advocacy and so on.

**Conclusions:**

Proactively addressing the array of public health challenges faced by rapidly growing ageing populations globally requires implementing collaborative, multisectoral policy solutions focused on promoting healthy, equitable, and socially engaged ageing. Healthcare systems, communities, and policies must be optimized to meet the needs of elderly people and tap into their strengths.

## 1 Introduction

The world's population is going through demographic change. With decreasing birth rates and longer life expectancies, populations across the globe are ageing rapidly. This shift presents challenges on a scale that require a holistic approach to meet the changing healthcare needs of these ageing populations. It is crucial to recognize and tackle the various public health obstacles that elderly people face.

It is estimated that by 2050, people aged 65 and above will surpass children under 5 years old worldwide for the first time in history [1]. According to the World Health Organization, the percentage of people globally over 60 years old in that same year will nearly double from 12% to 22% [2]. The ageing population will have effects on economies, social services, healthcare systems, communities and families. This significant demographic shift raises public health issues concerning chronic disease management, healthcare infrastructure and social support systems that demand immediate attention and innovative policy solutions [3].

Ageing brings about an increased vulnerability to illnesses, disabilities, geriatric conditions, mental health challenges and other ailments that necessitate enhanced medical care and long‐term services and support. Healthcare systems must be strengthened to address the increasing incidences of heart disease, cancer, diabetes, respiratory issues, cognitive decline, musculoskeletal disorders, frailty, incontinence and malnutrition [4]. Ageing not only involves these factors but also confronts challenges, such as ageism, social isolation, financial insecurity, elder mistreatment, caregiving responsibilities and obstacles in transportation, housing access and community engagement [5]. Urban settings and healthcare facilities tailored for the population may hinder ageing unless they incorporate age‐friendly adjustments.

This article delves into the health obstacles linked with ageing societies and proposes strategies to tackle the issues.

## 2 Literature Search Strategy

This narrative literature review synthesized research from public health, gerontology, geriatric medicine, social sciences and policy journals to summarize key public health challenges associated with ageing populations as well as evidence‐based policy responses.

A comprehensive search of literature was performed using the PubMed electronic database with the medical subject headings (MeSH) and combinations of terms including ‘ageing’, ‘aged’, ‘elderly’, ‘older adult’, ‘geriatrics’, ‘senior citizens’, ‘ageing population’, ‘public health’, ‘healthcare’, ‘health promotion’, ‘disease prevention’, ‘healthcare systems’, ‘health services’ and ‘health policy’. There were no limitations imposed on the date of publications; however, articles published since 2010 to 10 February 2024 were prioritized for relevance, but seminal older publications were also included. Grey literature reports on ageing policy and advocacy organization websites provided supplementary data to support our review. Non‐related records, non‐English manuscripts, personal opinions, duplicates, not relevant manuscripts, not originals and those not providing information concerning the earlier mentioned topics were excluded.

Data extracted from articles included details on specific public health issues and needs impacting seniors such as chronic conditions, disabilities, healthcare access barriers, caregiving demands, and social or environmental challenges. Policy recommendations or interventions described in the literature to address these public health needs were also extracted. These extracted data were synthesized into summary narratives describing key issues and policy responses in public health domains, including healthcare systems, communities and built environments, long‐term care, social supports, equity and rights.

A thematic analytical approach was applied to summarize the extracted data from the literature, and a number of important themes that emerged are discussed below.

## 3 Discussion of Key Results

### 3.1 Chronic Diseases in Ageing Populations

A major health concern linked to ageing populations is the occurrence of diseases [6, 7]. Chronic diseases, also referred to as non‐communicable diseases, are enduring health conditions that are typically not curable but can be managed through continuous medical care and lifestyle adjustments [8]. As individuals grow older, they are more prone to developing ailments such as heart disease, stroke, cancer, diabetes, arthritis, hypertension, dementia and lung disease [9]. According to the Centre for Disease Control, 6 of 10 adults in the United States have an illness and 30% of the adult population in the United States has at least one chronic condition [9]. The likelihood and frequency of illnesses increase significantly with age [10].

The substantial impact of disease on populations adversely affects their quality of life and functional abilities. Chronic conditions contribute to over 75% of healthcare expenditures for individuals aged 65 and above in the United States, amounting to over $1.5 trillion [11]. Common age‐related issues, such as frailty, falls and urinary incontinence, are also linked to having diseases [12].

The limitations in functioning and restricted activities caused by illnesses can result in a loss of independence, feelings of isolation and the necessity for ongoing care whether formal or informal [13].

For elderly individuals, managing chronic conditions is intricate and demands coordination among healthcare providers, integration of services, attention to potential drug interactions and encouragement for self‐management [14]. Nevertheless, the fragmentation and reimbursement policies of the healthcare systems linked to fee‐for‐service create barriers to delivering coordinated care tailored to patients with diseases [15].

An increasing challenge faced by the ageing population is the growing burden of cancer among individuals [16]. A heightened risk of developing cancer comes with ageing that allows mutations to accumulate over time in addition to exposure to carcinogens throughout a person's life [17] Ageing is a risk factor with about 80% of all cancer diagnoses in the United States made in individuals aged 55 years and above [18, 19].

As people get older, they face a risk of developing prostate, breast, lung and colorectal cancers due to the natural process of cellular ageing and the accumulation of carcinogens [20]. Dealing with cancer presents challenges. Elderly people often have other health issues that can complicate cancer treatments and make them harder to endure. For example, chemotherapy tends to cause side effects in elderly patients leading to adjustments in dosage or early treatment cessation, which can negatively impact outcomes [21]. Elderly patients also face increased risks with surgery, whereas radiotherapy and hormone therapy may be commonly used due to safety concerns. Moreover, fewer elderly people undergo cancer screening resulting in late‐stage diagnoses when the disease is more challenging to treat. These factors collectively contribute to morbidity and mortality rates for cancers among older adults compared to younger individuals [18]. Looking to the future, it is predicted that by 2030, there will be a 67% increase in new cases of cancer among elderly people [22].

Healthcare systems face obstacles when it comes to preventing cancer, conducting screenings, diagnosing the disease, providing efficient treatment, managing treatment side effects, offering end of life support and dealing with financial implications.

### 3.2 Preventable Morbidity and Injury in Ageing Populations

Major public health concerns for ageing populations are preventable morbidity and injury. Older adults are at high risk for many adverse health events that could be avoided or minimized through public health interventions. Key areas of preventable morbidity and injury include falls, adverse drug events, infections, pressure injuries, malnutrition, loneliness, suicide and abuse/exploitation.

Falls represent a significant marker of frailty, immobility and both acute and chronic health impairment in elderly individuals. Beyond the immediate physical injuries they cause, falls can lead to a cascade of adverse outcomes, including activity limitations, fear of falling and loss of mobility [23]. They are the leading cause of injury for those aged 65 and above, with millions requiring emergency care annually for fall injuries such as fractures, head traumas, and dislocations [24].

An area that can be overlooked but has been gathering momentum in the last few years is the impact of the menopause on women's health. As populations age, more and more women are experiencing the menopause and it can be considered a major life event that can have a detrimental impact on physical and mental health as well as on health issues in later life [25, 26]. It is anticipated that by 2025, the number of postmenopausal women will be around 1.1 billion worldwide and it is claimed that many will present with complex medical issues beyond the scope of traditional gynaecologists and general practitioners [27]. Not all women will experience short‐ or long‐term problems, but an increasing prevalence of osteoporosis, for example, leading to fractures, can be a particular issue [27].

There have been numerous studies about the menopause, including one ‘seeking to identify priorities and address unmet needs in clinical care, education, and access to treatment to improve quality of life for individuals during the menopause transition’ [28]. Quality of life can be affected during and after the menopause and be exacerbated by the ageing process [29]. After retrieving data from 21 other studies and 10 countries, Lambrinoudaki et al. [29] found that ‘the youngest age at menopause has been described as 48 years for women living in South Asia, the oldest as 52 years in Japanese women, and an intermediate age of 50 years for Caucasian women’. These and other studies show the need for educating health professionals, policymakers and women themselves about the menopause, along with the need to treat women in older age holistically as individuals going through this event. This approach will serve to enhance the health and quality of life of women in older age and thereby help limit the burdens on health systems.

Adverse drug events from medication errors, drug–drug interactions and inappropriate prescriptions also affect millions of older adults each year, often requiring hospital care [30]. Antibiotic‐resistant infections are also a major source of illness, as are influenza and pneumonia, due to weakened immunity [31]. Pressure injuries related to immobility affect up to 3 million elderly people annually [32]. Malnutrition impacts up to one‐third of the elderly that leads to an increase in morbidity [33].

### 3.3 Mental and Cognitive Health Challenges in Ageing Populations

Mental and cognitive health issues pose significant public health challenges as populations age. Common conditions such as depression, anxiety, dementia, delirium and substance abuse impact quality of life, functioning and healthcare utilization in older adults.

Depression affects up to 15% of older adults, is underdiagnosed and if left untreated, it can worsen outcomes of other conditions [34, 35, 36]. Anxiety disorders also worsen chronic disease and disability while reducing social engagement [37, 38]. Dementia cases, mainly Alzheimer's disease, are projected to triple by 2050, requiring extensive caregiving [39, 40]. Delirium, an acute confused state, affects over 7 million hospitalized elderly people annually, increasing morbidity and mortality [41, 42]. Alcohol and prescription drug abuse are also concerns for elderly populations and can exacerbate cognitive decline [43].

These conditions strain medical resources, caregivers and community services. Stigma around mental health issues can also prevent senior adults from seeking treatment [44].

### 3.4 Caregiving Gaps: Meeting the Needs of Ageing Populations

The growth of ageing populations worldwide places enormous strains on caregiving systems. Paid and unpaid caregivers are both in short supply, leading to major care gaps. This situation presents significant public health challenges.

Informal/family caregivers provide most of the long‐term care for elderly individuals, but this workforce is dwindling and overburdened [45]. Fewer family members are available to provide care due to declining birth rates and fragmented families. Home care workers are also in extremely short supply, leading to rationing of services and unmet needs [46]. Assisted living facilities and nursing homes face dire staffing shortages that impacts quality of care [47]. Even access to respite care and hospice/palliative services is inadequate.

These caregiving deficiencies lead to adverse outcomes, including caregiver stress and deterioration of health in elderly people. It is clear that building and sustaining an adequate caregiving workforce is imperative to enable well‐being, quality of life and dignity for ageing individuals. It presents daunting challenges but also provides opportunities to innovate systems and support.

### 3.5 Long‐Term Care Challenges for Ageing Populations

The rise in ageing of global populations means that long‐term care systems are facing serious challenges in meeting the increasing demand. Long‐term care refers to a range of medical, personal and social services required for chronic conditions and disability among older adults. Key long‐term care challenges include cost and affordability issues, geographic disparities, staffing difficulties, infrastructure deficits and discharge delays.

According to Genworth, the cost of long‐term care has been rising steadily, with nursing home care charges in the United States in 2021 averaging over $8910 per month. Limited public coverage places enormous financial strain on senior adults and their families. Rural areas also lack adequate long‐term care options leading to care access gaps based on geography [48]. Facilities nationwide face critical staffing shortages, impacting quality of care. Many nursing homes and other long‐term care settings have outdated infrastructure that is unsuited to today's care models.

### 3.6 Health Inequities in Ageing Populations

Health equity is a major public health challenge for elderly populations, with clear disparities along racial, ethnic, geographic and socioeconomic lines. This contributes to uneven opportunities for healthy ageing.

Racial and ethnic minorities often have higher rates of chronic disease, disability and mortality in later life due to the cumulative impacts of disadvantages [49]. Elderly people in rural areas frequently have reduced access to healthcare, community services and support compared to their urban counterparts, exacerbating rural health divides [50]. Low socioeconomic status among seniors also correlates to poorer health outcomes and barriers to care across settings [51]. Food and transportation insecurity make managing chronic conditions difficult for those with low income [52].

Closing these equity gaps requires improving healthcare access, economic support, community resources and environmental infrastructure in disadvantaged communities and populations. Achieving health equity is both a moral and public health imperative for our ageing society.

### 3.7 Healthcare Access Challenges for Ageing Populations

Key challenges for elderly people in accessing healthcare include transportation limitations, shortages of geriatric specialists and care fragmentation.

Mobility impairments and lack of transportation options impede the ability of elderly individuals to attend medical appointments, especially in rural or remote areas [53]. This leads to delayed care and poor health outcomes. Workforce shortages of geriatric specialists and primary care physicians trained in elder care also reduce access and quality of care [54]. While coping with possibly multiple chronic conditions, elderly people have to coordinate across many providers, but care fragmentation hampers this, resulting in conflicting treatment plans, medication issues and preventable deterioration [55].

Solutions to improve healthcare access for elderly individuals include expanding transportation services, funding more geriatric fellowships, integrating care teams, enhancing care transitions and investing in telehealth and outreach to bridge access gaps. With widespread population ageing, ensuring accessible, coordinated, high‐quality healthcare for seniors is an urgent priority.

### 3.8 End‐of‐Life Care Challenges for Ageing Populations

End‐of‐life care for rapidly growing ageing populations faces critical challenges around advanced care planning and place of death preferences versus realities.

Many seniors avoid making future care plans and having goals of care discussions with providers and families, leading to unwanted, aggressive interventions inconsistent with their wishes [56].

Additionally, although most elderly people prefer to die peacefully at home, the majority die in hospital often enduring painful, futile treatments [57]. This highlights the lack of hospice and palliative community infrastructure to enable home deaths.

Solutions involve promoting open conversations around care goals and normalizing hospice and advanced planning across healthcare. Clear advanced directives articulating preferences for medical treatments, resuscitation, proxies and hospice can prevent this. Additionally, policies and payment reforms must bolster community‐based palliative care to facilitate home deaths when desired. Honouring the voices and values of elderly people at the end of life is an ethical imperative.

### 3.9 Financial Safety Threats Facing Elderly Individuals

Financial and economic factors significantly impact health outcomes and well‐being for elderly people. Advancing age often brings heightened vulnerability to financial exploitation through technology abuse and ageism. Online scams targeting elderly people are rising rapidly, requiring strengthened consumer protections [58]. Common techniques include phishing emails, internet fraud and dishonest tech support schemes designed to steal identities, funds and personal information. Additionally, persistent ageist attitudes diminish the autonomy and self‐determination of elderly people while tacitly enabling mistreatment across settings [59]. Financial security and self‐determination require protection from those who prey on the unique vulnerabilities of elderly adults.

Key financial challenges include high out‐of‐pocket medical costs, prescription expenses, costly long‐term care, limited retirement income and resultant social isolation.

Out‐of‐pocket spending on deductibles, premiums and services not covered by Medicare or insurance create cost barriers to healthcare for many seniors [60]. Prescription medications for chronic conditions also impose financial hardship for those without adequate drug coverage. The exorbitant costs of long‐term care for nursing homes are unaffordable for most without insurance and declining pensions and inadequate retirement savings further reduce economic resources.

These financial burdens can deter elderly individuals from seeking healthcare and lead to skipped medications, inadequate nutrition and social isolation. Policy solutions such as expanding social programmes, reducing drug costs, creating affordable long‐term care options and strengthening retirement benefits are needed to address the economic barriers.

### 3.10 Built Environment Barriers Facing Elderly Population

The design of neighbourhoods, communities and homes often poses challenges to mobility, safety and well‐being. Age‐friendly modifications to housing and outdoor spaces are lacking.

Most existing homes and apartments were constructed without regard to limited mobility, creating hazards for ageing in places [61]. Features such as grab bars, ramps, chair lifts and lever‐style doors and faucets are rarely found in average housing. Without renovations, living at home can lead to slips, falls and injuries.

Additionally, public parks, streets and amenities are often designed without considering accessibility and usability. Detrimental built environments lacking sidewalks, parks and amenities reduce mobility and social connection [62]. Insufficient walking paths, benches, shade, recreational facilities and pedestrian safety features discourage elderly individuals from physical activity, social connections and community participation.

Promoting age‐friendly housing policies and community planning is imperative. Tax credits, zoning changes, universal design standards, housing vouchers, neighbourhood walkability initiatives and parks/centres tailored to the needs of elderly people are key to creating supportive environments for active ageing.

### 3.11 Safeguarding Rights in Ageing Populations

Advancing age often heightens the risks of basic human and civil rights being degraded or violated in multiple domains. Protecting elderly people from infringement of their fundamental freedoms and entitlements by unethical individuals, exploitative systems and discriminatory attitudes is an ongoing public health challenge. Elder abuse encompasses physical, psychological, sexual and financial abuses that violate basic human rights.

Ageism manifests itself across healthcare through the dismissal of concerns, inadequate pain management, priorities placed on younger patients and lack of access to care [63]. In long‐term care facilities, elder abuse through neglect, over‐medication and disrespect for dignity is far too common [64]. Societal prejudices diminish the autonomy of elderly people over their finances, healthcare decisions, sexuality and lifestyle choices [65].

Beyond physical health issues, many elderly individuals suffer from preventable issues such as loneliness, depression and social isolation that can lead to suicide in severe cases. Elder abuse is also a major public health crisis affecting up to 10% of elderly people. Public health measures aimed at screening, reporting, family supports and reducing ageism and social isolation are crucial.

Safeguarding senior citizens requires multifaceted public health approaches combining technology regulation, consumer education, reporting systems, social supports and campaigns to reduce the harm of ageism. Robust public health approaches must combine advocacy, education, improved complaint processes, legal reform, care oversight, anti‐ageism campaigns and infrastructure changes. The civil liberties and human rights of elderly people are urgent priorities as the mark of a just society is how it protects and empowers its most vulnerable members. Respect for the inherent dignity of all must remain paramount.

### 3.12 Climate Change and Sustainability Threats Facing Elderly Individuals

Climate change and environmental sustainability present multifaceted threats to the health and well‐being of ageing populations worldwide. seniors face disproportionate risks from heatwaves, natural disasters, changing disease patterns, food insecurity and climate anxiety.

The physiologic, chronic disease and social vulnerability profiles of many older adults increase their susceptibility to extreme heat events, which are rising due to climate change [66]. Elderly people are also especially vulnerable during climate‐exacerbated disasters such as floods, storms, droughts and wildfires in terms of mortality and displacement [67]. Shifting vector ecology is already altering geographic distributions of diseases impacting older adults, including West Nile, Lyme disease, malaria and dengue [68]. Food insecurity is also being amplified by climate disruptions, and older generations face grief and anxiety over the threats of climate change to the futures of their descendants [69].

Public health strategies to protect our ageing populations must integrate climate adaptation into emergency planning, disease surveillance, green infrastructure, resilient ageing services, sustainable supports and mental health responses. Environmental justice and sustainability for present and future elderly people remains an ethical priority.

### 3.13 Sociocultural Challenges Facing Ageing Populations

Ageing populations face diverse sociocultural challenges stemming from changing demographics, evolving social roles and supports and persistent ageism. Protecting the quality of life of elderly people requires addressing threats to social well‐being and cultural inclusion.

Shrinking family sizes and fragmented social networks reduce many social connections and care support [70]. Retirement or inability to work also changes community roles and purpose and ageist stereotypes in media and culture plus limited intergenerational interactions can socially isolate elderly people while denying their voices. Rapid adoption of advanced technology leaves many elderly people struggling with digital literacy and access barriers [71]. Accessibility of recreational, vocational, volunteer and learning programmes also lag.

Public health strategies to facilitate healthy ageing must combat ageism, boost social capital, integrate generations and adapt community services and spaces for changing demographics. Social and cultural inclusion of diverse senior citizens will grow even more crucial amid projected population ageing. Nurturing sociocultural support will require multidimensional policies, programs, urban planning and education to enable elderly people to continue their personal growth, purpose and sense of community.

### 3.14 Ageism and Elder Abuse: Threats to Dignity in Ageing

Ageism and elder abuse represent major public health threats impacting quality of life and dignity on a global scale. Ageism involves harmful stereotypes, attitudes and behaviours that marginalize, disrespect and disadvantage seniors based solely on their advanced age [72]. Ageist ideology manifests itself across multiple domains, including healthcare, housing, social services, the workplace, media representation, interpersonal interactions and policy decisions [73]. These prejudices can promote discrimination against the needs and autonomy of elderly people.

Closely tied to ageism, elder abuse encompasses physical, psychological sexual and financial abuses inflicting harm on senior citizens [59]. Neglect of basic needs within families and care facilities also constitutes abuse. Estimates suggest 10% or more of elderly people worldwide experience some form of abuse, causing physical and emotional trauma [74]. Like other forms of violence, elder abuse stems from power differentials as well as systemic and cultural forces.

To counteract these injustices, comprehensive public health approaches to education, reporting, policy reform, staff training and age‐friendly service provision are urgently needed. Fundamentally, cultivating a culture and systems that honour the value, voices, rights and dignity of people at all life stages represents an ethical imperative as populations increasingly ‘grey’. The worth of a society ultimately depends on how it treats its elders.

## 4 Addressing Public Health Challenges for Ageing Populations

Proactively addressing the myriad health and social challenges that have been outlined will be critical for protecting and enhancing the well‐being of elderly people. Although they may be complex, evidence‐based policy solutions implemented across sectors can help build more age‐friendly, equitable communities.

At the healthcare level, financing for elder care must be strengthened, geriatric training expanded, care systems integrated, technology leveraged judiciously and prevention prioritized. For example, meeting the complex cancer care needs of elderly patients requires coordinated, age‐friendly treatment approaches while managing comorbidities. Continued research on improving screening, therapies and delivery models is essential to reduce the rising cancer burden. Providing appropriate chronic disease treatment also necessitates innovations in team‐based care, health IT, telemedicine, care transitions, caregiver training and self‐management support. Recognition of the life‐changing impact that the menopause can have on women's health, particularly in older age, must help inform education and training of medical professionals, related health, social care and support workers. Women need access to evidence‐based information so they can take a full part in decisions around their own care including in later life.

Building liveable communities calls for accessible housing, transportation, recreational and green spaces to promote healthy behaviours and connections. Evidence‐based fall prevention programmes and age‐friendly home modifications could prevent many falls and related hospitalizations. Robust caregiver supports, respite services and adaptive workplace policies can help to reduce burdens. End‐of‐life care requires improved advanced planning guidance and community‐based hospice/palliative infrastructure.

Beyond clinical solutions, policies must counteract ageism and elder abuse through enhanced awareness, reporting and response mechanisms. Economic security issues necessitate shoring up of pensions, retirement benefits and safety nets. Promoting digital literacy and intergenerational exchange will help to keep elderly people socially engaged. Climate and emergency planning must specifically address the resilience needs of elderly people.

Finally, placing voices of elderly people at the decision‐making centre of policymaking, urban planning and resource allocation will ensure that responses match lived realities. Smart and collaborative public health approaches will enable communities to tap into the strengths and meet the needs of their ageing members. The generations we invest in today will shape our collective tomorrow.

## 5 Conclusion

Older populations face increased risks of disease, disability, geriatric conditions, climate change and disaster‐related threats, dementia and mental health challenges that strain healthcare systems primarily designed for care. Many of them will encounter challenges related to insecurity, lack of adequate caregiving support, age‐related discrimination (ageism), elder abuse concerns, limited transportation options, social isolation and menopause—all impacting well‐being and community engagement. Living environments in housing and in the locality often lack features that would enable inclusion and healthy ageing.

Although no single intervention alone can solve all the key issues highlighted above, implementing public health strategies across different sectors can provide a visionary and positive way forward that will provide benefits to communities now and in the future.

## Author Contributions

**Hafiz T. A. Khan**: Conceptualization, methodology and writing–original draft. **Kwaku Mari Addo**: Investigation, formal analysis and conceptualization. **Helen Findlay**: Writing–review and editing and conceptualization.

## Ethics Statement

The authors have nothing to report.

## Conflicts of Interest

The authors declare no conflicts of interest.

## Data Availability

The data that support the findings of this study are openly available in the University of West London repository at https://repository.uwl.ac.uk/.
